# Evaluation of the effect of methionine and glucosamine on adjuvant arthritis in rats

**DOI:** 10.3892/etm.2012.668

**Published:** 2012-08-16

**Authors:** YOSHIE YAMAGISHI, MAMORU IGARASHI, ATSUKO SUZUKI, SHIORI SUGURO, SHIN-ICHI HIRANO, ISAO NAGAOKA

**Affiliations:** 1 Protein Chemical Co. Ltd., Tokyo 100-0011;; 2 Department of Host Defense and Biochemical Research, Juntendo University, Graduate School of Medicine, Tokyo 113-8421;; 3 Mercian Cleantec Corporation, Fujisawa, Kanagawa 250-0057, Japan

**Keywords:** glucosamine, sulfur amino acid, methionine, adjuvant arthritis, rheumatoid arthritis

## Abstract

In the present study, we evaluated the effects of individual administration of methionine or glucosamine (GlcN) and compared with the combined administration of methionine and GlcN on the adjuvant arthritis model of rheumatoid arthritis in rats. Adjuvant arthritis was induced in female Lewis rats by injecting Freund’s complete adjuvant (FCA) into the right hind paws, and methionine (200 mg/kg body weight/day) and/or GlcN (400 mg/kg/day) were orally administered for 21 days. The progression of the adjuvant arthritis was clinically evaluated for characteristic signs and symptoms by employing an arthritis score. The administration of methionine combined with GlcN suppressed the swelling of FCA-uninjected left hind paws and the arthritis score. Additionally, histopathological examination revealed that the combined administration of methionine and GlcN markedly suppressed synovial hyperplasia and the destruction of the cartilage surface and articular meniscus of the knee joints of FCA-injected right hind paws. Furthermore, combined methionine and GlcN administration suppressed the increase in the levels of nitric oxide, prostaglandin E_2_ and hyaluronic acid in the plasma of rats with adjuvant arthritis. By contrast, individual administration of methionine or GlcN suppressed arthritis only slightly. These observations suggest that the combined administration of methionine and GlcN is more effective compared with individual administrations of methionine or GlcN in suppressing the progression of adjuvant arthritis (identified as swelling of joints and arthritis score), possibly by synergistically inhibiting synovial inflammation (identified as synovial hyperplasia and the destruction of the cartilage surface and articular meniscus) and the production of inflammatory mediators.

## Introduction

Rheumatoid arthritis affects approximately 1% of the general population and is characterized by the inflammatory propagation of synovial cells caused by articular injuries, resulting in an almost complete functional defect (1). Non-steroidal anti-inflammatory drugs (NSAIDs) are commonly used for the treatment of rheumatoid arthritis despite their gastric and renal toxicities (2). Although NSAIDs are efficient at reducing symptoms, including pain and edema, they have no effect on the basic disease process and do not protect against tissue or joint injury (3). Furthermore, NSAID treatment has been shown to enhance joint destruction in osteoarthritis (4) and inhibit the synthesis of glycosaminoglycans by articular chondrocytes (5).

Glucosamine (GlcN), a naturally occurring amino monosaccharide, is present in the connective and cartilage tissues and contributes to the maintenance of the strength, flexibility and elasticity of these tissues. GlcN has been widely used to treat osteoarthritis in humans (6). A number of short- and long-term clinical trials have demonstrated the significant symptom-modifying effects of GlcN in osteoarthritis (7–9). According to previous biochemical and pharmacological findings, the administration of GlcN normalizes cartilage metabolism by inhibiting degradation (10) and stimulating the synthesis of proteoglycans (11,12), and restores articular function. Additionally, GlcN has been reported to have an anti-inflammatory effect through inhibition of the production of inflammatory mediators, including nitric oxide (NO) and prostaglandin E_2_ (PGE_2_) (13).

Methylsulfonylmethane (MSM), a sulfur compound, is effective at treating osteoarthritis and, in combination with GlcN, demonstrates greater efficacy in the reduction of pain and swelling and the improvement of the functional ability of joints compared to the individual agents in humans (14). MSM also has an anti-inflammatory effect on type II collagen-induced arthritis in rats (15). Furthermore, MSM inhibits the lipopolysaccharide (LPS)-induced production of NO and PGE_2_ in the mouse macrophage-like cell RAW264.7 (16). Chondroitin sulfate, another sulfur compound, reduces pain, prevents narrowing of the knee joint space in humans (17) and inhibits the nuclear translocation of nuclear factor κB (NF-κB) in interleukin (IL)-1β-stimulated chondrocytes (18). Since sulfur compounds, such as MSM and chondroitin sulfate, have anti-inflammatory effects, methionine, a sulfur-containing amino acid, is also expected to exhibit an anti-inflammatory effect. However, no experimental data on the anti-inflammatory effects of methionine are currently available. Thus, in the present study, we evaluated the effect of methionine combined with GlcN on inflammation in the adjuvant arthritis model in rats.

## Materials and methods

### Animals

Female Lewis rats were purchased from Charles River Laboratories Japan, Inc. (Kanagawa, Japan). The animals were housed under specific pathogen-free conditions (controlled temperature of 24±3°C and humidity of 55±15%) and fed standard laboratory food and water *ad libitum*. To induce arthritis, rats were injected with Freund’s complete adjuvant (FCA), as described below. Animals received proper care and maintenance in accordance with institutional guidelines (Juntendo University, Graduate School of Medicine, Tokyo, Japan). The experiments adhered to the guidelines of the International Association for the Study of Pain (19).

### Induction of adjuvant arthritis

Adjuvant arthritis was induced in 8-week-old rats by a single intradermal injection of 0.1 ml FCA containing 0.5 mg heat-killed *M. tuberculosis* H37Ra emulsified in liquid paraffin (Wako Pure Chemical Industries, Osaka, Japan) into the footpad of the right hind paw (20,21).

Methionine (20 mg/ml) and GlcN (40 mg/ml; Protein Chemical Co., Ltd., Tokyo, Japan) were dissolved in 0.5% sodium carboxymethyl cellulose (CMC). Methionine and/or GlcN were orally administered by gavage to adjuvant-injected rats twice a day for 21 days at a dose of 200 mg/kg/day methionine and 400 mg/kg/day GlcN. As a vehicle control, 0.5% CMC solution was administered orally to rats with adjuvant arthritis instead of methionine or GlcN. Naïve control rats received orally administered 0.5% CMC, but were not injected with adjuvant. Six animals were used in each experimental group (naïve control, vehicle control, methionine, GlcN or methionine combined with GlcN).

### Evaluation of arthritis

The swelling of hind paws was monitored using a plethysmometer (TK-105, Muromachi Kikai Co., Ltd., Tokyo, Japan) prior to (day 0) and following (days 5, 8, 12, 15, 19 and 21) the FCA injection. The progression of adjuvant arthritis was clinically evaluated for the characteristic signs and symptoms, using an arthritis score that grades each paw from 0 to 4 points based on erythema and swelling of the joint (0 points, no erythema or swelling; 1 point, erythema or swelling of one toe; 2 points, erythema or swelling of two or more of the toes; 3 points, erythema and swelling of the entire paw; 4 points, complete erythema and swelling of the entire paw and an inability to bend the ankle) (21). Three paws, excluding the FCA-injected right hind paw (FCA-uninjected left hind paw and right and left fore paws) were scored and the highest possible score was 12.

### Histopathological evaluation of knee joints

Animals were sacrificed on day 22. The FCA-injected right and -uninjected left legs were resected above the ankle joints, and fixed in neutral 20% formalin. Following decalcification in formic acid, the knee joints were sectioned longitudinally and tissue sections (10 μm) were mounted on glass slides and stained with hematoxylin and eosin. Articular lesions were observed under a light microscope. Synovitis (synovial hyperplasia) was evaluated by measuring the area of the synovial membrane attached to the articular meniscus.

### Quantification of NO, PGE_2_ and hyaluronic acid (HA) in rat plasma

Blood samples were collected from the abdominal aorta under ether anesthesia on day 22. The heparin-anticogulated blood was centrifuged at 1500 x g for 10 min at 4°C to separate the plasma.

The total NO (nitrite and nitrate) level in the plasma was measured using a nitrate/nitrite colorimetric assay kit, according to the manufacturer’s instructions (Cayman Chemical Company, Ann Arbor, MI, USA). The sensitivity of the assay was <2.5 μM. The level of PGE_2_ in the plasma was measured using an enzyme-linked immunosorbent assay method, according to the manufacturer’s instructions (Cayman Chemical Company). The assay revealed no cross reactivity with other prostanoids, and the sensitivity was <15 pg/ml. HA levels in the plasma were measured using a hyaluronan assay kit, according to the manufacturer’s instructions (Seikagaku Biobusiness Corporation, Tokyo, Japan). The detection limit of the assay was 12.5 ng/ml.

### Statistical analysis

Statistical analyses of paw volume and arthritis score were performed using the Dunnett and Mann-Whitney U tests, respectively. Synovial membrane area and the levels of NO, PGE_2_ and HA were statistically analyzed using the Student’s t-test. Data were presented as the mean ± SE. P<0.05 was considered to indicate a statistically significant difference.

## Results

### Effects of methionine and GlcN on the inflammatory reaction in FCA-induced rat adjuvant arthritis

**Figure 1:**
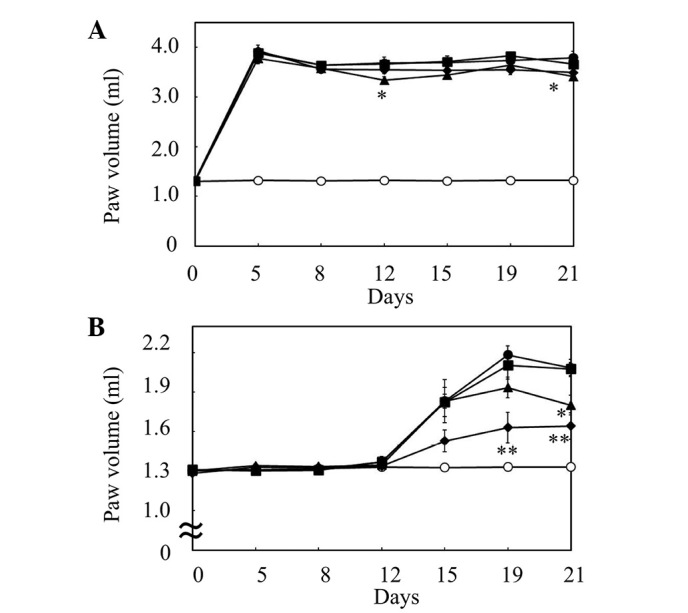
Effects of individual and combined administrations of methionine and GlcN on the swelling of hind paws in rat adjuvant arthritis. Adjuvant arthritis was induced by a single intradermal injection of FCA into the footpad of the right hind paw. Methionine (200 mg/kg/day; ▪), GlcN (400 mg/kg/day; ▴) or methionine combined with GlcN (♦) was administered orally for 21 days. Swelling of (A) FCA-injected right and (B) uninjected left hind paws was monitored using a plethysmometer before (day 0) and after (days 5, 8, 12, 15, 19 and 21) the FCA injection. As a vehicle control (•), 0.5% CMC solution was administered orally to rats with adjuvant arthritis instead of methionine or GlcN. Naïve control rats received orally administered 0.5% CMC, but were not injected with adjuvant (○). Data are the mean ± SE of six animals per experimental group. Values were compared between vehicle control and methionine, GlcN, or methionine combined with GlcN administration in adjuvant arthritis. ^*^P<0.05, ^**^P<0.01. GlcN, glucosamine; FCA, Freund’s complete adjuvant; CMC, carboxymethyl cellulose.

The swelling of hind paws was examined by measuring changes in paw volume. As shown in Fig. 1A, the swelling of the adjuvant-injected right hind paws increased rapidly after the injection, reached a maximum on day 5, and maintained an almost constant level until day 21 in the vehicle control rats. Administration of methionine and/or GlcN did not substantially change the swelling of adjuvant-injected right hind paws during the observation period, although GlcN administration slightly, but significantly, suppressed the swelling on days 12 and 21.

By contrast, the swelling of adjuvant-uninjected left hind paws gradually increased between days 12 and 21 (Fig. 1B). However, GlcN administration moderately suppressed the swelling (P<0.05) compared with the vehicle control. The administration of methionine combined with GlcN further suppressed the swelling on days 19 and 21 (P<0.01 compared with vehicle control). These observations suggest that the combined administration of methionine and GlcN suppresses the inflammatory reaction in FCA-induced adjuvant arthritis more efficiently compared with the single administration of methionine or GlcN.

### Effects of methionine and GlcN on an arthritis score in adjuvant arthritis

**Figure 2:**
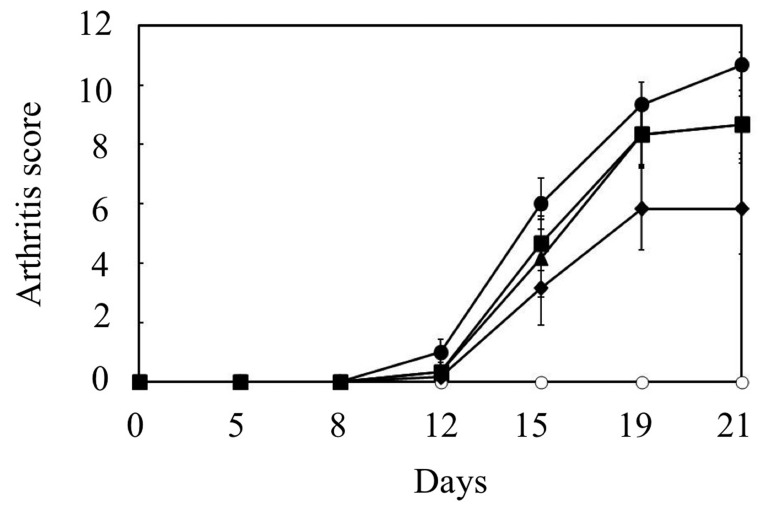
Effects of individual and combined administrations of methionine and GlcN on arthritis scores in rat adjuvant arthritis. Adjuvant arthritis was induced by a single intradermal injection into the footpad of the right hind paw. Methionine (200 mg/kg/day; ▪), GlcN (400 mg/kg/day; ▴) or methionine combined with GlcN (♦) was administered orally for 21 days. Arthritis was clinically evaluated using an arthritis score by grading each paw (excluding the FCA-injected right paw) from 0 to 4 points based on erythema and swelling of the joint before (day 0) and after (days 5, 8, 12, 15, 19 and 21) the FCA injection. As a vehicle control (•), 0.5% CMC solution was administered orally to rats with adjuvant arthritis instead of methionine or GlcN. Naïve control rats received orally administered 0.5% CMC, but were not injected with adjuvant (○). Data are the mean ± SE of six animals per experimental group. GlcN, glucosamine; FCA, Freund’s complete adjuvant; CMC, carboxymethyl cellulose.

The arthritis score was based on the severity and extent of erythema and swelling of the periarticular tissues, and the enlargement, distortion or ankylosis of the joints (21,22). In rats with adjuvant arthritis, the arthritis score increased progressively, and reached 11 points on day 21 (Fig. 2). Administration of methionine or GlcN slightly suppressed the increase in arthritis scores between days 12 and 21. Notably, the administration of methionine combined with GlcN further suppressed the increase in arthritis score between days 15 and 21, although the suppression was not statistically significant. Thus, the combined administration of methionine and GlcN more potently relieved the clinical signs and symptoms of adjuvant arthritis than individual administrations of methionine or GlcN.

### Effects of methionine and GlcN on histopathological changes in the joints in adjuvant arthritis

**Figure. 3.**
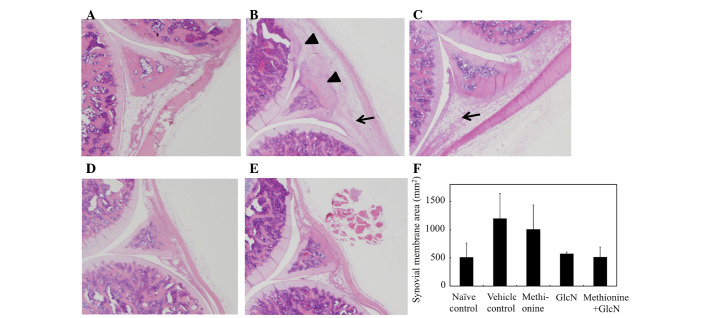
Effects of individual and combined administrations of methionine and GlcN on histopathological changes in FCA-injected right hind paw joints. Adjuvant arthritis was induced by a single intradermal injection of FCA into the footpad of the right hind paw. (C) Methionine (200 mg/kg/day), (D) GlcN (400 mg/kg/day or (E) methionine combined with GlcN was administered orally for 21 days. (B) As a vehicle control, 0.5% CMC solution was administered orally to rats with adjuvant arthritis instead of methionine or GlcN. (A) Naïve control rats received orally administered 0.5% CMC, but were not injected with adjuvant. On day 22, the right hind legs were resected, fixed and decalcified. The knee joints were longitudinally sectioned, and tissue sections (10 μm) were mounted on glass slides and stained with hematoxylin and eosin. Images are representative of 4 rats per group. (B) Synovial hyperplasia and destruction of the cartilage surface and articular meniscus are indicated by arrows and arrowheads, respectively. (F) Synovial hyperplasia was evaluated by measuring the area of the synovial membrane attached to the articular menisci using a K400 image analysis system. Data are the mean ± SE of four animals per experimental group. GlcN, glucosamine; FCA, Freund’s complete adjuvant; CMC, carboxymethyl cellulose.

Histopathological examination indicated that the cartilage surface and articular meniscus were destroyed by synovial hyperplasia in the knee joints of the FCA-injected right hind paws (Fig. 3B). Although synovial hyperplasia was observed, the destruction of the cartilage surface and articular meniscus was apparently suppressed in methionine-administered rats (Fig. 3C). Of note, the administration of GlcN or methionine combined with GlcN potently suppressed synovial hyperplasia and the destruction of the cartilage surface and articular meniscus in rats with adjuvant arthritis (Fig. 3D and E). The area of the synovial membrane was measured to evaluate the inflammatory reaction. The synovial membrane area was found to be increased by ∼2-fold in rats with adjuvant arthritis compared with naïve control rats (Fig. 3F). The administration of GlcN or methionine combined with GlcN notably suppressed the increase in synovial membrane area, whereas methionine administration only slightly abrogated the increase in synovial membrane area (Fig. 3F). These observations indicate that the administration of GlcN, or methionine combined with GlcN markedly suppressed the histopathological changes induced by inflammatory reactions in the knee joints of rats with adjuvant arthritis.

### Effects of methionine and GlcN on the plasma levels of NO, PGE_2_ and HA in adjuvant arthritis

**Figure. 4.**
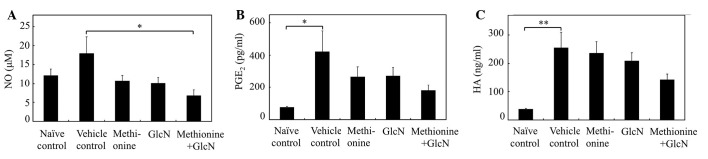
Effects of individual and combined administrations of methionine and GlcN on the plasma levels of NO, PGE_2_ and HA in rat adjuvant arthritis. Adjuvant arthritis was induced by a single intradermal injection of FCA into the footpad of the right hind paw. Methionine (200 mg/kg/day), GlcN (400 mg/kg/day) or methionine combined with GlcN was administered orally for 21 days. As a vehicle control, 0.5% CMC solution was administered orally to rats with adjuvant arthritis instead of methionine or GlcN. Naïve control rats received orally administered 0.5% CMC, but were not injected with adjuvant. On day 22, blood samples were collected for the preparation of plasma. (A) NO, (B) PGE_2_ and (C) HA levels in the plasma were measured using nitrate/nitrite colorimetric assay kit, an enzyme-linked immunosorbent PGE_2_ assay kit and hyaluronan assay kit, respectively. Data are the mean ± SE of six animals per experimental group. Values were compared between vehicle control and methionine, GlcN, or combined methionine and GlcN administration in rats with adjuvant arthritis. ^*^P<0.05, ^**^P<0.01. GlcN, glucosamine; NO, nitric oxide; PGE_2_, prostaglandin E_2_; HA, hyaluronic acid; FCA, Freund’s complete adjuvant; CMC, carboxymethyl cellulose.

NO is a gaseous-free radical which has been shown to be present at increased levels in the sera and synovial fluids of patients with rheumatoid arthritis (23). As shown in Fig. 4A, plasma NO levels in the rats with FCA-induced adjuvant arthritis were increased compared with the naïve control rats. Combined methionine and GlcN administration markedly reduced the levels of NO in the plasma of rats with adjuvant arthritis (P<0.05 compared with vehicle control rats).

Plasma PGE_2_ levels were elevated significantly in rats with FCA-induced adjuvant arthritis compared with naïve control rats (P<0.05; Fig. 4B). Combined methionine and GlcN administration markedly suppressed the increase in plasma PGE_2_ levels in rats with adjuvant arthritis, although the change was not significant (P<0.08). The HA level in the plasma was evaluated as a marker of synovial inflammation. HA levels were elevated significantly in rats with FCA-induced adjuvant arthritis compared with naïve control rats (P<0.01; Fig. 4C). Combined methionine and GlcN administration markedly suppressed the increase in plasma HA levels in rats with adjuvant arthritis. Thus, combined methionine and GlcN administration more potently suppressed the production of inflammatory mediators (NO and PGE_2_) and synovial inflammation (HA) in adjuvant arthritis compared with the individual administration of methionine or GlcN.

## Discussion

In the present study, we utilized adjuvant arthritis, a model of rheumatoid arthritis, to evaluate the effects of anti-inflammatory substances (20). Methionine, GlcN and a combination of the two were orally administered to rats with adjuvant arthritis, and the effects on the arthritis were microscopically and biochemically evaluated. Our results showed that the combined methionine and GlcN administration more potently suppressed not only the increase in swelling of the joints (Fig. 1) and arthritis score (Fig. 2), but also the histopathological changes in the joints in adjuvant arthritis (identified as synovial hyperplasia and destruction of cartilage surface and articular meniscus; Fig. 3). Furthermore, the combined methionine and GlcN administration markedly inhibited increases in the plasma levels of NO, PGE_2_ and HA in rats with adjuvant arthritis (Fig. 4). These observations suggest that the administration of a combination of methionine and GlcN is more effective than the individual administration of methionine or GlcN in suppressing the progression of adjuvant arthritis by inhibiting synovial inflammation and the production of inflammatory mediators.

The suppressive effects of GlcN on rat adjuvant arthritis have already been reported (24,25). Moreover, the administration of GlcN to patients with rheumatoid arthritis has been shown to significantly reduce the pain and swelling of arthritic joints compared with a placebo (26). Additionally, NO produced by synovial cells plays a role in the pathogenesis of rheumatoid arthritis (27), and PGE_2_ is also one of the important inflammatory mediators in rheumatoid arthritis (28). GlcN reportedly suppresses the IL-1β-mediated activation of synoviocytes, including IL-8, NO and PGE_2_, production (13). Although S-adenosylmethionine, a metabolite of methionine, has been demonstrated to be as effective as NSAIDs in the symptomatic management of osteoarthritis patients (29), the anti-inflammatory effect of methionine, one of the main sources of sulfur in the body, has not been reported. In the present study, we demonstrated that methionine slightly suppressed the progression of adjuvant arthritis by inhibiting the inflammatory reaction and the production of inflammatory mediators. Thus, the combination of methionine and GlcN is likely to exert a synergistic effect on adjuvant arthritis, possibly by suppressing synovial inflammation and the production of inflammatory mediators.

Additionally, we preliminarily evaluated the effect of cystine (200 mg/kg), a sulfur amino acid, on adjuvant arthritis. Cystine demonstrated suppressive effects on adjuvant arthritis to the same extent as methionine; i.e., cystine suppressed the swelling of the FCA-injected right and -uninjected left paws, the arthritis score, the histopathological changes in joints, NO and PGE_2_ production and synovial inflammation (HA level; data not shown). However, the combination of cystine and GlcN revealed no inhibitory effect on adjuvant arthritis, although the combination of methionine and GlcN potently suppressed adjuvant arthritis, as demonstrated in the present study. These observations suggest that methionine and cystine act on the adjuvant arthritis, possibly through different mechanisms, despite the fact that methionine and cystine are classified as sulfur amino acids.

In summary, the administration of methionine to rats with adjuvant arthritis slightly suppressed the arthritis. However, the combined administration of methionine and GlcN markedly suppressed the arthritis, possibly by a synergistic effect, thereby inhibiting synovial inflammation and the production of inflammatory mediators in adjuvant arthritis.
